# Pre-exposure to *Candida glabrata* protects *Galleria mellonella* against subsequent lethal fungal infections

**DOI:** 10.1080/21505594.2020.1848107

**Published:** 2020-11-29

**Authors:** Xiao-Wen Huang, Mei-Nian Xu, Huan-Xin Zheng, Meng-Lei Wang, Li Li, Kang Zeng, De-Dong Li

**Affiliations:** a Department of Dermatology, Nanfang Hospital, Southern Medical University, Guangzhou, China; b Division of Rheumatology and Clinical Immunology, Department of Medicine, University of Pittsburgh, Pittsburgh, PA, USA; c Central Laboratory, Shanghai Skin Disease Hospital, Tongji University School of Medicine, Shanghai, China

**Keywords:** *Candida glabrata*, *Galleria mellonella*, innate immunity, hemocytes, immune priming

## Abstract

Commensal fungi are an important part of human microbial community, among which *Candida albicans* and *Candida glabrata* are two common opportunistic pathogens. Unlike the high pathogenicity of *C. albicans, C. glabrata* is reported to show low pathogenicity to the host. Here, by using a *Galleria mellonella* infection model, we were able to confirm the much lower virulence of *C. glabrata* than *C. albicans*. Interestingly, pre-exposure to live *C. glabrata* (LCG) protects the larvae against subsequent various lethal fungal infections, including *C. albicans, Candida tropicalis,* and *Cryptococcus neoformans*. Inconsistently, heat-inactivated *C. glabrata* (HICG) pre-exposure can only protect against *C. albicans* or *C. tropicalis* re-infection, but not *C. neoformans*. Mechanistically, LCG or HICG pre-exposure enhanced the fungicidal activity of hemocytes against *C. albicans* or *C. tropicalis*. Meanwhile, LCG pre-exposure enhanced the humoral immunity by modulating the expression of fungal defending proteins in the cell-free hemolymph, which may contribute to the protection against *C. neoformans*. Together, this study suggests the important role of *C. glabrata* in enhancing host immunity, and demonstrates the great potential of *G. mellonella* model in studying the innate immune responses against infections.

## Introduction

The mammalian harbors enormous commensal microbes, including bacteria, fungi, and viruses, etc., which are considered to be closely related to the health of the host [1]. In the past decades, more and more studies demonstrated the connection between mammalian commensal bacteria and host immunity [2]. These commensal bacteria are reported to shape both local and systemic immune responses and are related to many diseases such as inflammatory bowel disease (IBD), diabetes, and tumors, etc. [2,3]. However, as another important component of the mammalian microflora, the impact of symbiotic fungi on the host immunity has not been well revealed.

*Candida* species are the most common commensal fungi in the human intestine, with *Candida albicans* and *Candida glabrata* are the two ubiquitous strains that can be found in most healthy humans [4]. Recently, it was reported that the commensal *C. albicans* might be able to recapitulate the protective benefits of intestinal bacteria by calibrating the responsiveness of circulating immune cells [5]. Moreover, *C. albicans* and its cell wall ligand β-glucan are reported to induce functional reprogramming of innate immune cells, resulting in resistance of the host to subsequent lethal infections [6,7]. The protection induced by *C. albicans* relies on the memory of host innate immune cells and is called “Trained Immunity” [8]. Different from the extensive research on *C. albicans*, the effect of *C. glabrata* on the host immunity is still unknown.

*Galleria mellonella* is an emerging invertebrate model to study the pathogenicity of microorganisms. Unlike mammals, *G. mellonella* lacks adaptive immunity and its innate immune system mainly relies on the cellular immunity of hemocytes and the humoral immunity of cell-free hemolymph [9]. Hemocytes are the main phagocytes of the larvae, which are functional equivalents of mammalian macrophages [10]. Considering the unique immune system, *G. mellonella* has the potential to be used for studying the innate immune memory. Indeed, it was reported that pre-exposure of the larvae of *G. mellonella* to a sub-lethal dose of *C. albicans* or *S. cerevisiae* protects against subsequent lethal *C. albicans* infection [11]. The enhanced immune responses of *G. mellonella* were also observed after *Aspergillus fumigatus*, bacterium, microbial cell wall components, or even stress (physical or thermal) priming [12–15]. This enhanced innate immune responses in *G. mellonella* is called “Immune Priming”, which is considered to be an important survival strategy for insects [16–18].

Unlike the high virulence of *C. albicans, C. glabrata* shows low pathogenicity in both mouse and *G. mellonella* models [19–21]. Whether the low-virulence *C. glabrata* would enhance the host immunity against other infections is unknown yet. Here, by using the *G. mellonella* model, we evaluated the protective effects of *C. glabrata* against subsequent lethal fungal infections caused by *C. albicans, Candida tropicalis,* or *Cryptococcus neoformans*. Also, the modulation of *C. glabrata* to the cellular immunity and humoral immunity of *G. mellonella* was explored.

## Results

### Prior exposure to C. glabrata decreases the susceptibility of larvae to subsequent lethal fungal infections

*C. glabrata* was reported to be less virulent than *C. albicans* in mouse as well as *G. mellonella* [20,21]. In this study, we first evaluated the pathogenicity of a clinical *C. glabrata* isolate GH15016 in *G. mellonella*. Consistent with previous report [21], our results showed that *C. glabrata* GH15016 showed much lower virulence in *G. mellonella*. As shown in Figure S1, 1 × 10 ^6^ CFUs of *C. glabrata* cannot kill *G. mellonella* within 12 days of observation, while 5 × 10 ^5^ CFUs of *C. albicans* SC5314 caused all the larvae died within 2 days.

**Figure 1.**
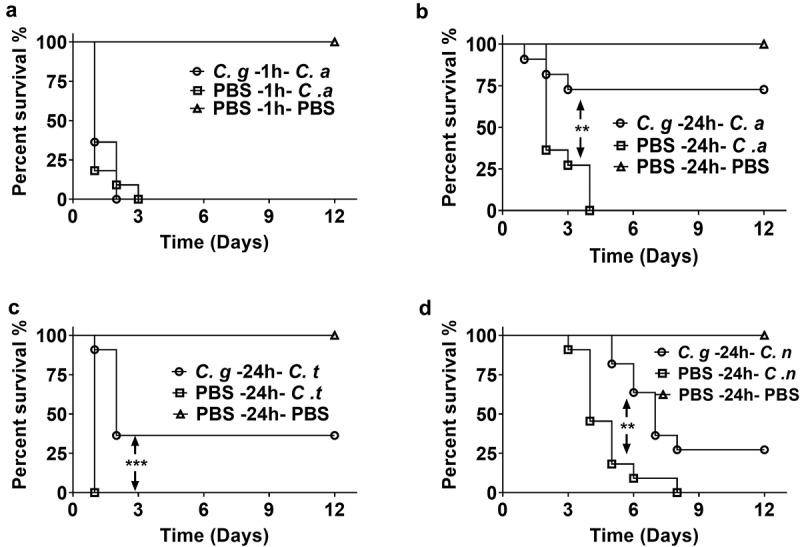
**Survival curves of *G. mellonella* infection with different fungi**. (a) *G. mellonella* larvae were injected with 5 × 10 ^5^ CFUs of *C. albicans* 1 h post 1 × 10 ^5^ CFUs of *C. glabrata* infection. (b, c, d) *G. mellonella* were injected with 1 × 10 ^5^ CFUs of *C. glabrata* or PBS, after 24 h, the larvae were infected with 5 × 10 ^5^ CFUs of *C. albicans* (b), 1 × 10 ^6^ CFUs of *C. tropicalis* (c) or 1 × 10 ^5^ CFUs of *C. neoformans* (d). Each group contained 11 randomly chosen larvae. Differences were determined by using the log-rank test.* *P* < 0.05. ** *P* < 0.01. *** *P* < 0.001

Next, we asked whether the exposure to *C. glabrata* can affect the susceptibility of the larvae to other fungal infections. First, *G. mellonella* were infected with 1 × 10 ^5^ CFUs of *C. glabrata*. 1 h post *C. glabrata* infection, the larvae were infected with a lethal dose of *C. albicans*, and the survival was observed. The results showed that 1 h pre-exposure to *C. glabrata* did not affect the susceptibility of *G. mellonella* to *C. albicans*, as all the larvae died within 3 days, similar to the survival curve of *C. albicans* mono-infection group (Figure 1(a)). Then we adjusted the infection strategy by injecting *C. glabrata* 24 h before *C. albicans* infection. Interestingly, 24 h prior exposure to *C. glabrata* significantly improved the survival rate of infected larvae. As shown in Figure 1(b), all larvae in the 5 × 10 ^5^ CFUs *C. albicans* infection group succumbed within 3 days, while over 75% larvae pre-exposed to 1 × 10 ^5^ CFUs of *C. glabrata* survived within 12 days of observation. To test whether the protection is *C. albicans* specific or not, other pathogenic fungi including *C. tropicalis* and *C. neoformans* were used for the secondary infection. And the protective effects were found for both pathogens (Figure 1(c,d)). These results indicated that pre-exposure to *C. glabrata* protects *G. mellonella* from subsequent various lethal fungal infections.

**Figure 2.**
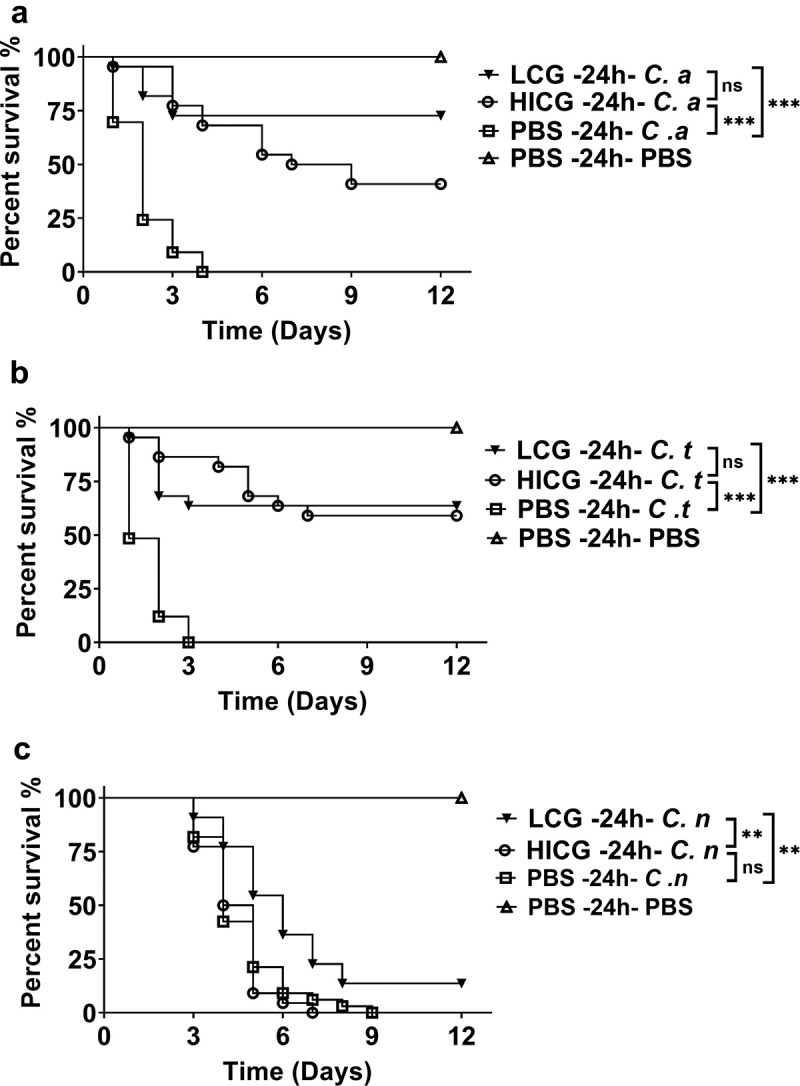
**Survival curves of *G. mellonella* infection with different fungi post live *C. glabrata* (LCG) or heat-inactivated *C. glabrata* (HICG) exposure**. *G. mellonella* were injected with 1 × 10 ^5^ CFUs of LCG, HICG or PBS, 24 h later the larvae were re-injected with 5 × 10 ^5^ CFUs of *C. albicans* (a), 1 × 10 ^6^ CFUs of *C. tropicalis* (b), or 1 × 10 ^5^ CFUs of *C. neoformans* (c). The experiments were repeated 3 times independently. Differences were determined by using the log-rank test. * *P* < 0.05. ** *P* < 0.01. *** *P* < 0.001

In order to explore the mechanism by which *C. glabrata* triggered the protective immune responses of *G. mellonella*, heat-inactivated *C. glabrata* (HICG) or *C. glabrata* supernatant were used to evaluate the protective effects. As shown in Figure 2(a,b), pre-exposure to HICG was able to protect *G. mellonella* against subsequent *C. albicans* or *C. tropicalis* infection, and there is no significant difference in the protective effects between HICG and LCG. Surprisingly, HICG failed to protect the larvae against *C. neoformans* re-infection (Figure 2(c)). Moreover, the supernatant of *C. glabrata* did not show a protective effect against any secondary infection (data not shown).

### C. glabrata exposure enhanced the cellular immunity of G. mellonella

**Figure 3.**
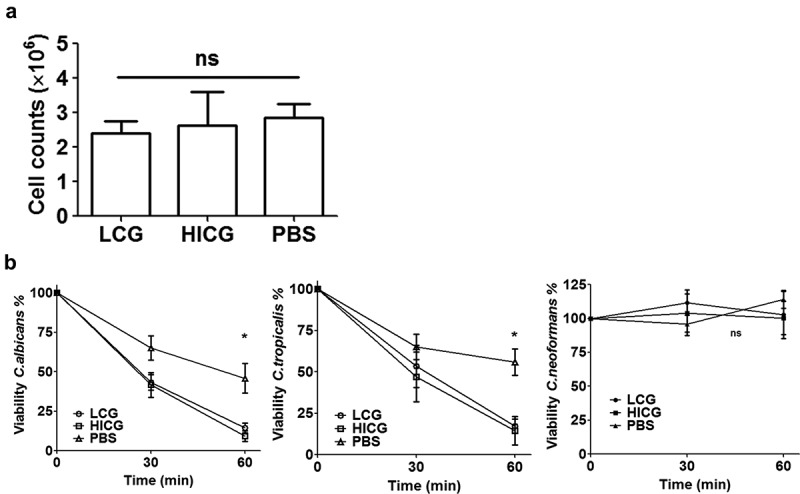
**Changes of cellular immunity of *G. mellonella* post live *C. glabrata* (LCG) or heat-inactivated *C. glabrata* (HICG) exposure**. (a) The number of hemocytes in the cell-free hemolymph after the larvae were exposed with 1 × 10 ^5^ CFUs of LCG or HICG for 24 h. (b) Killing activity of hemocytes against *C. albicans, C. tropicalis*, or *C. neoformans*. Larvae were inoculated with 1 × 10 ^5^ CFUs of LCG or HICG for 24 h, and then hemocytes were harvested for assessing the fungicidal activity. The experiments were repeated 3 times independently. Results are shown as mean ± SEM and statistical analysis was performed by One-way ANOVA. * *P* < 0.05

To clarify the mechanisms behind the protection of *C. glabrata* pre-exposure, the number of hemocytes in the hemolymph of larvae 24 h post *C. glabrata* priming was counted. As shown in Figure 3(a), after the larvae were exposed to 1 × 10 ^5^ CFUs of LCG or HICG for 24 h, no significant changes in the number of hemocytes was found. However, *C. glabrata* exposure significantly enhanced the fungicidal activity of hemocytes against *C. albicans* and *C. tropicalis*. As shown in Figure 3(b), co-incubation of hemocytes with *C. albicans* or *C. tropicalis* resulted in approximately 50% death of the strains within 60 minutes, while *C. glabrata* pre-exposure significantly reduced the survival rate of strain to less than 20%. Strikingly, hemocytes failed to kill *C. neoformans*, regardless of the pre-exposure to *C. glabrata* or not (Figure 3(b)). Even prolonging the incubation time to up to 4 h could not promote the clearance of *C. neoformans* by hemocytes (data not shown). These results indicated that the cellular immune responses that mainly rely on the hemocytes of the larvae are involved in the clearance of *C. albicans* and *C. tropicalis*, but not *C. neoformans*.

### C. glabrata exposure triggered the proteome changes of G. mellonella cell-free hemolymph

**Figure 4.**
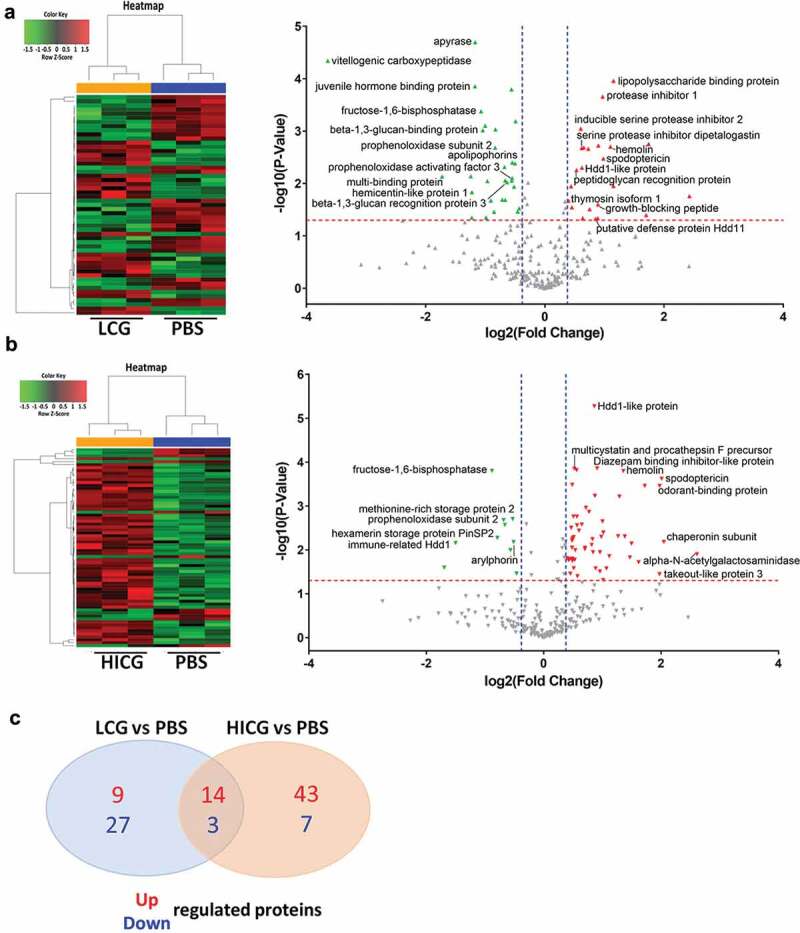
**Proteomics analysis of *G. mellonella* cell-free hemolymph after exposure of larvae to live *C. glabrata* (LCG) or heat-inactivated *C. glabrata* (HICG)**. (a, b) Left: Unsupervised hierarchical clusters of *G. mellonella* cell-free hemolymph protein profiles in groups of LCG versus PBS (a), and HICG versus PBS (b). Right: Volcano plots to distinguish the differentially expressed proteins filtered by a fold change of more than 1.3 or less than 0.77 and a maximum *P-value* of 0.05. (c) Intersection and union of differentially expressed proteins between LCG versus PBS group and HICG versus PBS group

The *G. mellonella* immune system consists of interconnected cellular and humoral responses. Since our results showed that the cellular immunity did not contribute to the host defense against *C. neoformans* infection, we further evaluated the humoral immunity of the larvae in response to *C. glabrata* exposure. SWATH-MS-based quantitative proteomics analysis was performed on the *G. mellonella* cell-free hemolymph after exposure of the larvae to LCG (1 × 10 ^5^ CFUs/Larva), HICG (1 × 10 ^5^ CFUs/Larva), or PBS for 24 h. In total, 369 proteins were identified. We first compared the proteome differences between LCG infection group and PBS control group. As shown in Figure 4(a) and Table S1, 53 proteins were determined to be differentially abundant (*P* < 0.05) with a fold change of more than 1.3 or less than 0.77. In detail, 23 proteins were up-regulated 24 h post-LCG infection, including lipopolysaccharide-binding protein, hemolin, spodoptericin, protease inhibitor 1, growth-blocking peptide, putative defense protein Hdd11, Hdd1-like protein, serine protease inhibitor dipetalogastin, inducible serine protease inhibitor 2, peptidoglycan recognition protein, and thymosin isoform 1, etc., and 30 proteins were down-regulated, such as vitellogenic carboxypeptidase, apyrase, hemicentin-like protein 1, juvenile hormone-binding protein, beta-1,3-glucan-binding protein, beta-1,3-glucan recognition protein 3, fructose-1,6-bisphosphatase, prophenoloxidase subunit 2, multi-binding protein, prophenoloxidase activating factor 3, and apolipophorins, etc. (Figure 4(a) and Table S1).

Also, we analyzed the proteome changes of cell-free hemolymph 24 h after the larvae were infected with HICG (1 × 10 ^5^ CFUs/Larva). The protein profile exhibited 67 differentially expressed proteins, of which 57 proteins were up-regulated and 10 proteins were down-regulated. Many of the proteins are involved in proteolysis, nitrogen compound metabolic process, and the regulation of innate immune responses. Among the up-regulated proteins, alpha-N-acetylgalactosaminidase, chaperonin subunit, spodoptericin, odorant-binding protein, takeout-like protein 3, Hdd1-like protein, diazepam binding inhibitor-like protein, multicystatin and procathepsin F precursor, and hemolin, etc., showed the most significant expression difference (Figure 4(b) and Table S2). In contrast, fructose-1,6-bisphosphatase, methionine-rich storage protein 2, prophenoloxidase subunit 2, hexamerin storage protein PinSP2, arylphorin, and immune-related Hdd1, etc., were highly down-regulated after HICG exposure (Figure 4(b) and Table S2).

Since both LCG and HICG pre-exposure can protect *G. mellonella* against subsequent *C. albicans* or *C. tropicalis* infection, we extracted the common differentially expressed proteins from these two sets of data. As shown in Figure 4(c) and Table 1, 17 proteins showed the same expression pattern after exposure to LCG or HICG, of which 14 proteins were up-regulated, and 3 proteins were down-regulated. Most of the up-regulated proteins are related to pathogen recognition and innate immune responses. For example, peptidoglycan recognition protein is considered to be related to the identification of pathogens in insects [22]. Hemolin, spodoptericin, and Hdd1-like protein are associated with innate immune responses. Protease inhibitor 1, serine protease inhibitor dipetalogast, and inducible serine protease inhibitor 2 were active against various toxic proteases released by fungus [23]. Among the three down-regulated proteins, prophenoloxidase subunit 2 has the oxidoreductase activity, while apolipophorin is related to the defense against the Gram-positive bacteria *L.monocytogenes* [24,25]. Changes in the expression of these proteins might contribute to the protection against *C. albicans* or *C. tropicalis* infection.

**Table 1.** Proteins that showed the same expression trend after exposure to live *C. glabrata* (LCG) or heat-inactivated *C. glabrata* (HICG) exposure

	Proteins	GO analysis	LCG vs. PBS		HICG vs. PBS	
			*P*-value ^#^	FC ^&^	*P*-value ^#^	FC ^&^
Up-reuglated	odorant-binding protein	odorant binding	**	3.34	***	3.93
	lipopolysaccharide binding protein	sugar binding	***	2.22	***	1.40
	hemolin	innate immune response	***	2.18	***	2.41
	spodoptericin	innate immune response	**	1.97	***	4.03
	protease inhibitor 1	peptidase inhibitor activity	***	1.96	***	3.30
	AGAP004366-PA	proline biosynthetic process	**	1.86	**	1.97
	growth-blocking peptide	growth factor activity	*	1.85	**	2.02
	Hdd1-like protein	innate immune response	**	1.54	***	1.82
	Serine protease inhibitor dipetalogast	endopeptidase inhibitor activity	**	1.53	**	2.00
	Inducible serine protease inhibitor 2	peptidase inhibitor activity	***	1.52	*	1.79
	peptidoglycan recognition protein	innate immune response	**	1.44	***	1.83
	27 kDa hemolymph protein		*	1.37	***	1.55
	arginine kinase	arginine kinase activity;	*	1.36	**	1.52
	thymosin isoform 1	immune response	*	1.32	**	1.57
Down-regulated	Apolipophorins	lipid transporter activity	*	0.70	*	0.73
	prophenoloxidase subunit 2	oxygen transporter activity	**	0.56	**	0.62
	fructose-1,6-bisphosphatase	phosphoric ester hydrolase activity	***	0.48	***	0.54

^#^: * *P* < 0.05, ** *P* < 0.01, *** *P* < 0.001;

^&:^FC: Fold Change.

**Figure 5.**
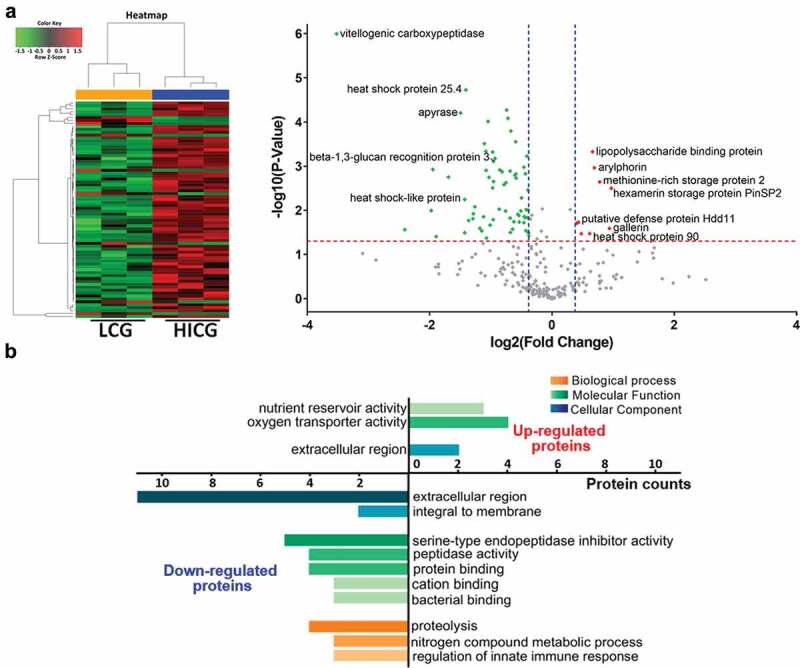
**Proteome differences of *G. mellonella* cell-free hemolymph after live *C. glabrata* (LCG) or heat-inactivated *C. glabrata* (HICG) exposure**. (a) Left: Unsupervised hierarchical clusters of *G. mellonella* cell-free hemolymph protein profiles post LCG exposure or HICG exposure. Right: Volcano plots to distinguish the differentially expressed proteins filtered by a fold change of more than 1.3 or less than 0.77 and a maximum *P-value* of 0.05. (b) Bar chart representing GO terms for biological process, molecular function, and cellular component enriched in the up-regulated and down-regulated proteins of LCG exposure group versus HICG exposure group

To further determine the differences of humoral immune responses between LCG exposure group and HICG exposure group, we compared the proteome between the two groups directly. As shown in Figure 5(a) and Table S3, compared with HICG exposure, LCG priming caused 74 differentially expressed proteins in the cell-free hemolymph, of which 9 proteins were up-regulated, and 65 proteins were down-regulated. The up-regulated proteins include hexamerin storage protein PinSP2, arylphorin, lipopolysaccharide-binding protein, heat shock protein 90, putative defense protein Hdd11, methionine-rich storage protein 2, and gallerin, etc., while the down-regulated proteins include vitellogenic carboxypeptidase, heat shock protein 25.4, apyrase, beta-1,3-glucan recognition protein 3, and heat shock-like protein, etc. Gene Ontology (GO) analysis was performed to identify items for biological process, molecular function, and cellular component enriched in proteins of these two groups. As shown in Figure 5(b), 4 of the nine up-regulated proteins showed oxygen transporter activities, while three showed nutrient reservoir activities. Among the down-regulated proteins, five proteins showed serine-type endopeptidase inhibitor activities and four proteins are involved in the proteolysis process. These differentially expressed proteins underline the difference in humoral immune responses of *G. mellonella* to LCG or HICG exposure and might contribute to the protection against *C. neoformans* infection.

## Discussion

*C. glabrata* is an opportunistic fungal pathogen as well as a common commensal fungus colonized in human intestine. Here, by using a *G. mellonella* infection model, we were able to confirm the lower virulence of *C. glabrata* than *C. albicans*. Interestingly, we found that pre-exposure to *C. glabrata* protects the larvae against subsequent lethal fungal infections, including *C. albicans, C. tropicalis,* and *C. neoformans*. Our results further showed that *C. glabrata* pre-exposure enhances the fungicidal activity of hemocytes against *C. albicans* and *C. tropicalis*, while they cannot effectively kill *C. neoformans*. Instead, pre-exposure to LCG enhanced the humoral immunity of cell-free hemolymph by up-regulating the expression of defense proteins and redox related proteins, etc., which may contribute to the protection against *C. neoformans*.

Our results showed that both LCG and HICG priming could enhance the fungicidal activity of hemocytes as well as protect *G. mellonella* against subsequent *C. albicans* or *C. tropicalis* infection, suggesting cell wall components might be accountable for the protection. In line with our results, it was reported that *C. albicans* and its cell wall β-glucan are able to induce functional reprogramming of monocytes and macrophages, leading to enhanced protection against infections caused by fungi or protozoans [6,7]. Besides β-glucan, another highly conserved ligand of *Candida* species mannans, is also reported to enhance the host defense against *C. albicans* or viral infections [5,11]. Considering the similar protective capacity of LCG and HICG against subsequent *C. albicans* or *C. tropicalis* infection, the protective effects might be mainly mediated by *C. glabrata* cell wall ligands, through the activation of hemocytes.

Besides the enhancing fungicidal activity of hemocytes, the results of the proteomic analysis showed that both LCG and HICG exposure could affect the protein expression in cell-free hemolymph of *G. mellonella*. Proteins up-regulated in both groups are mainly related to fungal recognition and defense. E.g. the main function of peptidoglycan recognition proteins in insects is to recognize pathogens [22], and their up-regulation may induce secretion of pro-inflammatory mediators [26]. Hemolin is an immunoglobulin-like protein exclusively found in Lepidoptera, which functions as an opsonin in defense against pathogens in *G. mellonella* [27]. Hdd11 is the *G. mellonella* homologue of Noduler, which can bind to yeast β-1,3-glucan and traps microorganisms and hemocytes into the nodule, then kill the pathogens [28]. Spodoptericin is a defensin-like antimicrobial peptide (AMP) [29], while lipopolysaccharide-binding protein can enhance the activity of AMP [30]. Thymosin exerts immunomodulatory effects on the silkworm *Bombyx mori*, rendering them resistant to viral infection [31]. The up-regulation of these proteins indicated that humoral immunity is also involved in the protection against subsequent *Candida* infection.

Here, we found that hemocytes failed to kill *C. neoformans* in *G. mellonella*, which is in line with previous reports that *C. neoformans* can evade macrophage attack [32,33]. Also, it was reported that although the hemocytes of *G. mellonella* can phagocytose *C. neoformans*, it does not translate into effectively fungal cell clearance, and as low as 20 fungal cells of *C. neoformans* were able to kill caterpillars [34]. The inability of hemocytes to clear *C. neoformans* may be related to the yeast’s adaptation to an intracellular lifestyle. Indeed, upon phagocytosis, *C. neoformans* can undergo morphological changes, such as capsular enlargement, fungal giant cell formation, cell to cell spread, and nonlytic exocytosis [35]. These changes may help *C. neoformans* use these phagocytes as shelters for invasion and proliferation [32,33].

The results showed that though LCG is able to protect the larvae against subsequent *C. neoformans* infection, the level of protection is less than that against *C. albicans* or *C. tropicalis*. It is not surprising because LCG pre-exposure significantly enhanced the fungicidal activity of hemocytes against *C. albicans* and *C. tropicalis*. However, hemocytes cannot effectively clear *C. neoformans*, even after LCG priming. Considering LCG but not HICG can protect the larvae against *C. neoformans* infection, we speculate that unknown ligands released by LCG activate the humoral immunity of *G. mellonella*, and promote the clearance of *C. neoformans*. By comparing the hemolymph proteome between the LCG exposure group and the HICG exposure group, we were able to determine the different effects of these two treatments on the humoral immune responses of the larvae. Although the unknown function of many differential proteins limits the interpretation of the results, we could still find some interesting proteins. For example, putative defense protein Hdd11 was up-regulated in LCG exposure group. Since nodulation is the predominant insect immune response to bacterial and fungal infections [36], the up-regulation of putative defense protein Hdd11 might contribute to the defense. In addition, differences in the expression of several heat shock proteins after LCG or HICG priming indicated different oxidative stress states of the larvae.

Our results showed that *C. glabrata* pre-exposure protected *G. mellonella* from subsequent fungal infections, which is reminiscent of the concept of “Immune Priming” [18]. “Immune Priming” is an enhanced innate immune response that exists in invertebrates which do not depend on the adaptive immune system. For example, pre-infection with a tapeworm *Schistocephalus solidus* can protect the copepod *Macrocyclops albidus* against subsequent similar re-infection [37]. Also, *Lactobacillus* species can modulate the immune system of *G. mellonella* and protect against subsequent infections [38,39]. These innate immune responses in invertebrates might be mediated by the macrophage-like innate immune cells hemocytes [40]. “Immune Priming” is often compared with the concept of “Trained Immunity” [8,18,41], which is an innate immune memory in vertebrates. “Trained Immunity” is first discovered to be primed by *C. albicans* and its cell wall β-glucan [6]. It is independent of adaptive immunity but relies on the long-term functional reprogramming of innate immune cells, mainly monocytes and macrophages [41]. Although these two types of immune responses have high similarities, there are still many unclear points. For example, “Trained Immunity” results in the long-term reprogramming of host immune cells and usually have a long-term protective effect. However, many of the reported “Immune Priming” cases in invertebrates might be due to a sustained immune response from the heterologous or intervening challenge [16]. Here, we found that 24-hour pre-infection of *C. glabrata* can protect the larvae from subsequent lethal fungal infections. Whether this protection can last longer time or has transgenerational effects need to be figured out in the future.

Overall, our results showed that pre-exposure to *C. glabrata* could protect *G. mellonella* against subsequent fungal infections, which might be contributed by the increased fungicidal activity of hemocytes and the enhanced humoral immunity in hemolymph. This study suggests the important role of *C. glabrata* in enhancing host immunity, as well as the wide application of the *G. mellonella* model in the field of immunology.

## Materials and methods

### Strains and growth conditions

*C. albicans* reference strain SC5314, *C. neoformans* reference strain H99, clinical *C. glabrata* isolate GH15016, and clinical *C. tropicalis* isolate 8915 were used in this study. All strains were routinely grown in YPD (1% yeast extract, 2% peptone and 2% dextrose) liquid medium at 35°C in a shaking incubator. All the clinical isolates were provided by Microbiology Laboratory of PLA General Hospital, Beijing, China. For the preparation of heat-inactivated strains, the cultures were inoculated for 60 min at 70°C and washed with PBS. The resultant pellet was sampled and inoculated onto YPD solid medium to verify that the strains had been killed.

### G. mellonella killing assay

The *G. mellonella* infection model was carried out using previously described protocols [42]. Briefly, final instar stage *G. mellonella* larvae (Wax Moth Breeding Inc., Tianjin, China) with the appropriate weight (250 ± 25 mg) were selected. The inoculum was injected in a 5 μl volume directly to the last, left pro-leg using a Hamilton syringe [43]. The second injection was delivered to the last right pro-leg if needed. A mock inoculation with PBS was performed in each experiment to monitor killing due to physical injury or infection by pathogenic contaminants. Each group contained 11 randomly chosen larvae. After injection, larvae were incubated at 37°C and the number of dead larvae was scored daily.

### Quantification of G. mellonella hemocyte

*G. mellonella* hemocyte quantification was performed by using a protocol from Rossoni et al. [38]. Briefly, 24 h after *C. glabrata* strain GH15016 infection, four larvae per group were bled into pre-chilled micro-centrifuge tubes to prevent melanization. The collected hemolymph was diluted in cold, sterile insect physiologic saline (IPS) (150 mM sodium chloride; 5 mM potassium chloride; 100 mM Tris-HCl, pH 6.9 with 10 mM EDTA, and 30 mM sodium citrate). The hemocytes were identified based on cell morphology and quantified using a hemocytometer.

### Determination of the fungicidal activity of hemocytes

Fungicidal activity of hemocytes was determined using a protocol from Sheehan et al. [44] with slight modification. Briefly, 24 h after *C. glabrata* strain GH15016 infection, larvae were bled into cold IPS. Hemocytes were extracted by centrifuging and incubated at a density of 2 × 10 ^5^ cells/ml at 30°C in PBS in a rapidly stirred chamber. Cell-free hemolymph opsonized strains were added with the final concentration of 2 × 10 ^6^ CFU/ml. The killing was measured by diluting and plating cell suspensions onto YPD solid medium after incubation for different minutes. Colony counts were performed in triplicate for each sample and the mean values were obtained.

### Proteomics analysis of larval hemolymph

After *G. mellonella* were infected with LCG, HICG, or PBS for 24 h, the abdomen of the larvae was cut open and hemocytes were removed by centrifugation for 10 minutes. The cell-free hemolymph was diluted in PBS and the proteins were quantified by the Bradford protein assay. Each group has three independent samples. The proteins were reduced with dithiothreitol (DTT), alkylated with iodoacetamide (IAA) and digested with trypsin [45]. Protein identification was performed using MaxQuant v1.5.2.8 quantitative proteomics software (www.maxquant.org/), and label-free quantification (LFQ) normalization was performed using Perseus v.1.5.6.0 software package (www.maxquant.org/). Proteins with a fold change of more than 1.3 or less than 0.77, as well as *P* < 0.05, were determined to be differentially abundant and statistically significant. Protein identification results were shown in Table S4. For comparing the proteome differences between LCG infection group, HICG infection group, and PBS control group, hierarchical clustering was generating by R software v.3.5.0 (www.r-project.org/) and Volcano maps were created by GraphPad Prism. GO enrichment analysis was then performed to gain insight into the underlying biological processes, molecular function, and cellular component related to the differentially expressed proteins in LCG infection group vs. HICG infection group.

### Statistical analysis

All experiments were repeated independently in triplicate. Killing curves were plotted and examined using the Kaplan-Meier method, and differences were determined using the log-rank test. Hemocyte counts were shown as mean ± SEM and statistical analysis was performed by One-way ANOVA. The results of the fungicidal activity of hemocytes were expressed as a percentage of the original number at time zero and shown as mean ± SEM. Statistical significance in the fungicidal activity of hemocytes was assessed by One-way ANOVA. Statistical analysis was performed using GraphPad Prism. A *P-value* of 0.05 in all replicate experiments was considered statistically significant.

## Supplementary Material

Supplemental Material
