# Supplementary material for: Pre-exposure to Candida glabrata protects Galleria mellonella against subsequent lethal fungal infections
Source: Virulence. 2020 Nov 29;11(1):1674–84. doi: 10.1080/21505594.2020.1848107 (PMC7714416; doi:10.1080/21505594.2020.1848107)
Supplement: Supplemental Material [file KVIR_A_1848107_SM7405.zip › Figure S1 caption.docx]

**Figure S1.** **Survival rates of *G. mellonella* infection with *C. glabrata* GH15016 or *C. albicans* SC5314.** The larvae were infected with different concentrations of *C. glabrata* or *C. albicans* and incubated at 37 °C.
