# Supplementary material for: Pre-exposure to Candida glabrata protects Galleria mellonella against subsequent lethal fungal infections
Source: Virulence. 2020 Nov 29;11(1):1674–84. doi: 10.1080/21505594.2020.1848107 (PMC7714416; doi:10.1080/21505594.2020.1848107)
Supplement: Supplemental Material [file KVIR_A_1848107_SM7405.zip › Table 1.docx]

Table 1. Proteins that showed the same expression trend after exposure to live *C. glabrata* (LCG) or heat-inactivated *C. glabrata* (HICG) exposure.

|  | Proteins | GO analysis | LCG vs. PBS | | HICG vs. PBS | |
| --- | --- | --- | --- | --- | --- | --- |
|  |  |  | *P*-value ^#^ | FC ^&^ | *P*-value ^#^ | FC ^&^ |
| Up-reuglated | odorant-binding protein | odorant binding | ** | 3.34 | *** | 3.93 |
|  | lipopolysaccharide binding protein | sugar binding | *** | 2.22 | *** | 1.40 |
|  | hemolin | innate immune response | *** | 2.18 | *** | 2.41 |
|  | spodoptericin | innate immune response | ** | 1.97 | *** | 4.03 |
|  | protease inhibitor 1 | peptidase inhibitor activity | *** | 1.96 | *** | 3.30 |
|  | AGAP004366-PA | proline biosynthetic process | ** | 1.86 | ** | 1.97 |
|  | growth-blocking peptide | growth factor activity | * | 1.85 | ** | 2.02 |
|  | Hdd1-like protein | innate immune response | ** | 1.54 | *** | 1.82 |
|  | Serine protease inhibitor dipetalogast | endopeptidase inhibitor activity | ** | 1.53 | ** | 2.00 |
|  | Inducible serine protease inhibitor 2 | peptidase inhibitor activity | *** | 1.52 | * | 1.79 |
|  | peptidoglycan recognition protein | innate immune response | ** | 1.44 | *** | 1.83 |
|  | 27 kDa hemolymph protein |  | * | 1.37 | *** | 1.55 |
|  | arginine kinase | arginine kinase activity; | * | 1.36 | ** | 1.52 |
|  | thymosin isoform 1 | immune response | * | 1.32 | ** | 1.57 |
| Down-regulated | Apolipophorins | lipid transporter activity | * | 0.70 | * | 0.73 |
|  | prophenoloxidase subunit 2 | oxygen transporter activity | ** | 0.56 | ** | 0.62 |
|  | fructose-1,6-bisphosphatase | phosphoric ester hydrolase activity | *** | 0.48 | *** | 0.54 |

^#^: * *P* < 0.05, ** *P* < 0.01, *** *P* < 0.001;

^&^: FC: Fold Change
