# Supplementary material for: Pre-exposure to Candida glabrata protects Galleria mellonella against subsequent lethal fungal infections
Source: Virulence. 2020 Nov 29;11(1):1674–84. doi: 10.1080/21505594.2020.1848107 (PMC7714416; doi:10.1080/21505594.2020.1848107)
Supplement: Supplemental Material [file KVIR_A_1848107_SM7405.zip › Table S1.docx]

Table S1. Dysregulated proteins in *G. mellonella* cell-free hemolymph after exposure of larvae to live *C. glabrata* (LCG) versus PBS for 24 h.

|  | Proteins | LCG vs. PBS | |
| --- | --- | --- | --- |
|  |  | p-value ^#^ | FC ^&^ |
| Up-regulated | similar to CG10638-PA | * | 5.37 |
|  | odorant-binding protein | ** | 3.34 |
|  | similar to predicted protein | * | 3.25 |
|  | AGAP002157-PA | * | 2.22 |
|  | lipopolysaccharide binding protein | *** | 2.22 |
|  | hemolin | ** | 2.14 |
|  | spodoptericin | ** | 1.97 |
|  | protease inhibitor 1 | *** | 1.96 |
|  | AGAP004366-PA | ** | 1.86 |
|  | growth-blocking peptide | * | 1.85 |
|  | putative defense protein Hdd11 | * | 1.85 |
|  | hexamerin storage srotein PinSP1 | * | 1.80 |
|  | gallerin | * | 1.68 |
|  | yellow5 | ** | 1.66 |
|  | arylphorin | ** | 1.57 |
|  | 14-3-3zeta | * | 1.55 |
|  | Hdd1-like protein | ** | 1.54 |
|  | serine protease inhibitor dipetalogastin | ** | 1.53 |
|  | inducible serine protease inhibitor 2 | *** | 1.52 |
|  | peptidoglycan recognition protein | ** | 1.44 |
|  | 27 kDa hemolymph protein | * | 1.37 |
|  | arginine kinase | * | 1.36 |
|  | thymosin isoform 1 | * | 1.32 |
| Down-regulated | hypothetical protein | * | 0.74 |
|  | abnormal wing disc-like protein | * | 0.73 |
|  | similar to prolylcarboxypeptidase | *** | 0.71 |
|  | apolipophorins | ** | 0.71 |
|  | unknown | * | 0.70 |
|  | aminoacylase | ** | 0.68 |
|  | prophenoloxidase activating factor 3 | ** | 0.68 |
|  | beta-1,3-glucan recognition protein precursor | ** | 0.68 |
|  | promoting protein | *** | 0.68 |
|  | beta-1,3-glucan recognition protein 3 | ** | 0.65 |
|  | hypothetical protein AaeL_AAEL010802 | ** | 0.63 |
|  | laminin | * | 0.63 |
|  | BmP109 | ** | 0.63 |
|  | serpin 13 | * | 0.61 |
|  | hypothetical protein TNAV2c_gp132 | *** | 0.56 |
|  | prophenoloxidase subunit 2 | ** | 0.56 |
|  | triacylglycerol lipase | * | 0.55 |
|  | CALNUC | * | 0.53 |
|  | hemicentin, putative | ** | 0.51 |
|  | heat shock-like protein | * | 0.50 |
|  | yellow4 | *** | 0.50 |
|  | beta-1,3-glucan-binding protein | *** | 0.49 |
|  | fructose-1,6-bisphosphatase | *** | 0.48 |
|  | apyrase | *** | 0.45 |
|  | juvenile hormone binding protein | *** | 0.44 |
|  | hemicentin-like protein 1 | * | 0.43 |
|  | hypothetical protein BRAFLDRAFT_228127 | * | 0.43 |
|  | 32 kDa ferritin subunit | ** | 0.42 |
|  | multi-binding protein | ** | 0.30 |
|  | vitellogenic carboxypeptidase | *** | 0.08 |

^#^ * *P* < 0.05, ** *P* < 0.01, *** *P* < 0.001;

^&^ FC: Fold Change
