# Supplementary material for: Pre-exposure to Candida glabrata protects Galleria mellonella against subsequent lethal fungal infections
Source: Virulence. 2020 Nov 29;11(1):1674–84. doi: 10.1080/21505594.2020.1848107 (PMC7714416; doi:10.1080/21505594.2020.1848107)
Supplement: Supplemental Material [file KVIR_A_1848107_SM7405.zip › Table S2.docx]

Table S2. Dysregulated proteins in G. mellonella cell-free hemolymph after exposure of larvae to heat-inactivated *C. glabrata* (HICG) versus PBS for 24 hours.

|  | Proteins | HICG vs. PBS | |
| --- | --- | --- | --- |
|  |  | p-value ^#^ | FC ^&^ |
| Up-regulated | alpha-N-acetylgalactosaminidase | * | 6.11 |
|  | chaperonin subunit, putative | ** | 4.13 |
|  | spodoptericin | *** | 4.03 |
|  | odorant-binding protein | *** | 3.93 |
|  | takeout-like protein 3 | * | 3.93 |
|  | protease inhibitor 1 | *** | 3.30 |
|  | fumarylacetoacetase | * | 3.07 |
|  | hemicentin-like protein 2 | ** | 2.82 |
|  | hypothetical protein BRAFLDRAFT_90416 | * | 2.76 |
|  | Kunitz-like protease inhibitor precursor | ** | 2.61 |
|  | hemolin | *** | 2.56 |
|  | BCP inhibitor | *** | 2.44 |
|  | hypothetical protein | ** | 2.41 |
|  | anionic antimicrobial peptide 2 | * | 2.17 |
|  | cobatoxin-like protein | * | 2.10 |
|  | growth-blocking peptide | ** | 2.02 |
|  | inducible metalloproteinase inhibitor protein | * | 2.02 |
|  | serine protease inhibitor dipetalogastin | ** | 2.00 |
|  | AGAP004366-PA | ** | 1.97 |
|  | pattern recognition serine proteinase precursor | * | 1.95 |
|  | beta-1,3-glucan recognition protein 3 | * | 1.94 |
|  | Diazepam binding inhibitor-like protein | *** | 1.88 |
|  | chemosensory protein | * | 1.85 |
|  | peptidoglycan recognition protein | *** | 1.83 |
|  | Hdd1-like protein | *** | 1.82 |
|  | inducible serine protease inhibitor 2 | * | 1.79 |
|  | heat shock protein 25.4 | ** | 1.78 |
|  | gloverin-like protein | ** | 1.76 |
|  | unknown | ** | 1.71 |
|  | scolexin | ** | 1.65 |
|  | sulfatase | ** | 1.63 |
|  | cecropin-A | ** | 1.63 |
|  | thymosin isoform 1 | ** | 1.57 |
|  | arginine kinase | ** | 1.52 |
|  | conserved hypothetical protein | * | 1.49 |
|  | diapause bioclock protein | ** | 1.48 |
|  | Gelsolin | ** | 1.48 |
|  | yellow1 | *** | 1.48 |
|  | heat shock protein hsp21.4 | * | 1.45 |
|  | prophenoloxidase activating factor 3 | * | 1.44 |
|  | multicystatin and procathepsin F precursor | *** | 1.44 |
|  | AChain A | * | 1.43 |
|  | peptidoglycan recognition-like protein B | ** | 1.42 |
|  | 27 kDa hemolymph protein | ** | 1.42 |
|  | cationic peptide CP8 precursor | ** | 1.41 |
|  | juvenile hormone binding protein | * | 1.40 |
|  | peptidylprolyl isomerase B | * | 1.40 |
|  | lipopolysaccharide binding protein | *** | 1.40 |
|  | similar to GA18153-PA | ** | 1.40 |
|  | BmP109 | ** | 1.40 |
|  | imaginal disc growth factor | ** | 1.40 |
|  | imaginal disc growth factor-like protein | * | 1.39 |
|  | chemosensory protein 11 | ** | 1.38 |
|  | similar to apolipoprotein D | * | 1.37 |
|  | heat shock-like protein | * | 1.36 |
|  | lacunin | * | 1.34 |
|  | transferrin precursor | * | 1.32 |
| Down-regulated | apolipophorins | * | 0.73 |
|  | arylphorin | ** | 0.70 |
|  | methionine-rich storage protein 2 | ** | 0.69 |
|  | carboxylesterase-6 | * | 0.68 |
|  | methionine-rich storage protein | ** | 0.63 |
|  | prophenoloxidase subunit 2 | ** | 0.62 |
|  | hexamerin storage protein PinSP2 | ** | 0.58 |
|  | fructose-1,6-bisphosphatase | *** | 0.54 |
|  | immune-related Hdd1 | ** | 0.35 |
|  | death-related protein | * | 0.31 |

^#^ * *P* < 0.05, ** *P* < 0.01, *** *P* < 0.001;

^&^ FC: Fold Change
