# Supplementary material for: Pre-exposure to Candida glabrata protects Galleria mellonella against subsequent lethal fungal infections
Source: Virulence. 2020 Nov 29;11(1):1674–84. doi: 10.1080/21505594.2020.1848107 (PMC7714416; doi:10.1080/21505594.2020.1848107)
Supplement: Supplemental Material [file KVIR_A_1848107_SM7405.zip › Table S3.docx]

Table S3. Dysregulated proteins in *G. mellonella* cell-free hemolymph after exposure of larvae to live *C. glabrata* (LCG) versus heat-inactivated *C. glabrata* (HICG) for 24 h.

|  | Proteins | LCG vs. HICG | |
| --- | --- | --- | --- |
|  |  | p-value ^#^ | FC ^&^ |
| Up-regulated | hexamerin storage protein PinSP2 | ** | 1.96 |
|  | gallerin | * | 1.92 |
|  | methionine-rich storage protein 2 | ** | 1.72 |
|  | arylphorin | ** | 1.62 |
|  | lipopolysaccharide binding protein | *** | 1.59 |
|  | methionine-rich storage protein | * | 1.54 |
|  | heat shock protein 90 | * | 1.40 |
|  | carboxylesterase-6 | * | 1.35 |
|  | putative defense protein Hdd11 | * | 1.32 |
|  | serine protease inhibitor dipetalogastin | * | 0.77 |
| Down-regulated | adhesion-like transmembrane protein | ** | 0.76 |
|  | putative serine protease-like protein 2 | ** | 0.76 |
|  | cationic peptide CP8 precursor | * | 0.76 |
|  | AGAP010733-PA | *** | 0.75 |
|  | similar to prolylcarboxypeptidase | ** | 0.75 |
|  | abnormal wing disc-like protein | * | 0.74 |
|  | similar to apolipoprotein D | ** | 0.74 |
|  | serpin 11 | * | 0.74 |
|  | 26kDa ferritin subunit | * | 0.73 |
|  | imaginal disc growth factor-like protein | * | 0.73 |
|  | peptidoglycan recognition-like protein B | ** | 0.73 |
|  | diapause bioclock protein | ** | 0.71 |
|  | carboxylesterase | * | 0.70 |
|  | cyclophilin-like protein | * | 0.69 |
|  | hypothetical protein AaeL_AAEL010802 | * | 0.69 |
|  | Diazepam binding inhibitor-like protein | * | 0.68 |
|  | imaginal disc growth factor | ** | 0.67 |
|  | juvenile hormone binding protein | ** | 0.66 |
|  | twelve cysteine protein 1 | *** | 0.66 |
|  | 27 kDa hemolymph protein | ** | 0.66 |
|  | AChain A | * | 0.65 |
|  | similar to GA18153-PA | * | 0.64 |
|  | yellow1 | *** | 0.63 |
|  | GK19979 | * | 0.63 |
|  | similar to CG10638-PA | * | 0.62 |
|  | hypothetical protein | ** | 0.62 |
|  | chemosensory protein 11 | *** | 0.61 |
|  | chemosensory protein | *** | 0.60 |
|  | protease inhibitor 1 | ** | 0.59 |
|  | lacunin | * | 0.59 |
|  | peptidylprolyl isomerase B | ** | 0.58 |
|  | BmP109 | ** | 0.57 |
|  | hypothetical protein TNAV2c_gp132 | ** | 0.55 |
|  | similar to collagen, type XI, alpha 1 isoform 2 | ** | 0.55 |
|  | beta-1,3-glucan recognition protein precursor | *** | 0.52 |
|  | beta-1,3-glucan-binding protein | ** | 0.52 |
|  | beta-1,3-glucan recognition protein 3 | *** | 0.52 |
|  | kazal-type proteinase inhibitor | * | 0.51 |
|  | yellow4 | *** | 0.51 |
|  | triacylglycerol lipase | ** | 0.51 |
|  | peptidoglycan recognition protein | * | 0.50 |
|  | spodoptericin | ** | 0.49 |
|  | unknown | *** | 0.48 |
|  | fatty acid-binding protein 1 | * | 0.48 |
|  | prophenoloxidase activating factor 3 | ** | 0.47 |
|  | BCP inhibitor | *** | 0.47 |
|  | scolexin | *** | 0.47 |
|  | cecropin-A | * | 0.46 |
|  | putative mitochondrial Mn superoxide dismutase | * | 0.45 |
|  | anionic antimicrobial peptide 2 | * | 0.44 |
|  | odorant binding protein | ** | 0.42 |
|  | hemicentin-like protein 2 | * | 0.41 |
|  | chaperonin subunit, putative | * | 0.41 |
|  | similar to vacuolar ATP synthase subunit S1 | * | 0.39 |
|  | heat shock protein 25.4 | *** | 0.38 |
|  | heat shock-like protein | ** | 0.37 |
|  | GG20702 | * | 0.37 |
|  | apyrase | *** | 0.36 |
|  | putative secreted peptide 30 | ** | 0.31 |
|  | alpha-N-acetylgalactosaminidase | * | 0.27 |
|  | multi-binding protein | ** | 0.26 |
|  | glucosidase 2 subunit beta precursor, putative | * | 0.25 |
|  | takeout-like protein 3 | * | 0.19 |
|  | vitellogenic carboxypeptidase | *** | 0.09 |

^#^ * *P* < 0.05, ** *P* < 0.01, *** *P* < 0.001;

^&^ FC: Fold Change
