# Supplementary figures and images for: Pre-exposure to Candida glabrata protects Galleria mellonella against subsequent lethal fungal infections
Source: Virulence. 2020 Nov 29;11(1):1674–84. doi: 10.1080/21505594.2020.1848107 (PMC7714416; doi:10.1080/21505594.2020.1848107)

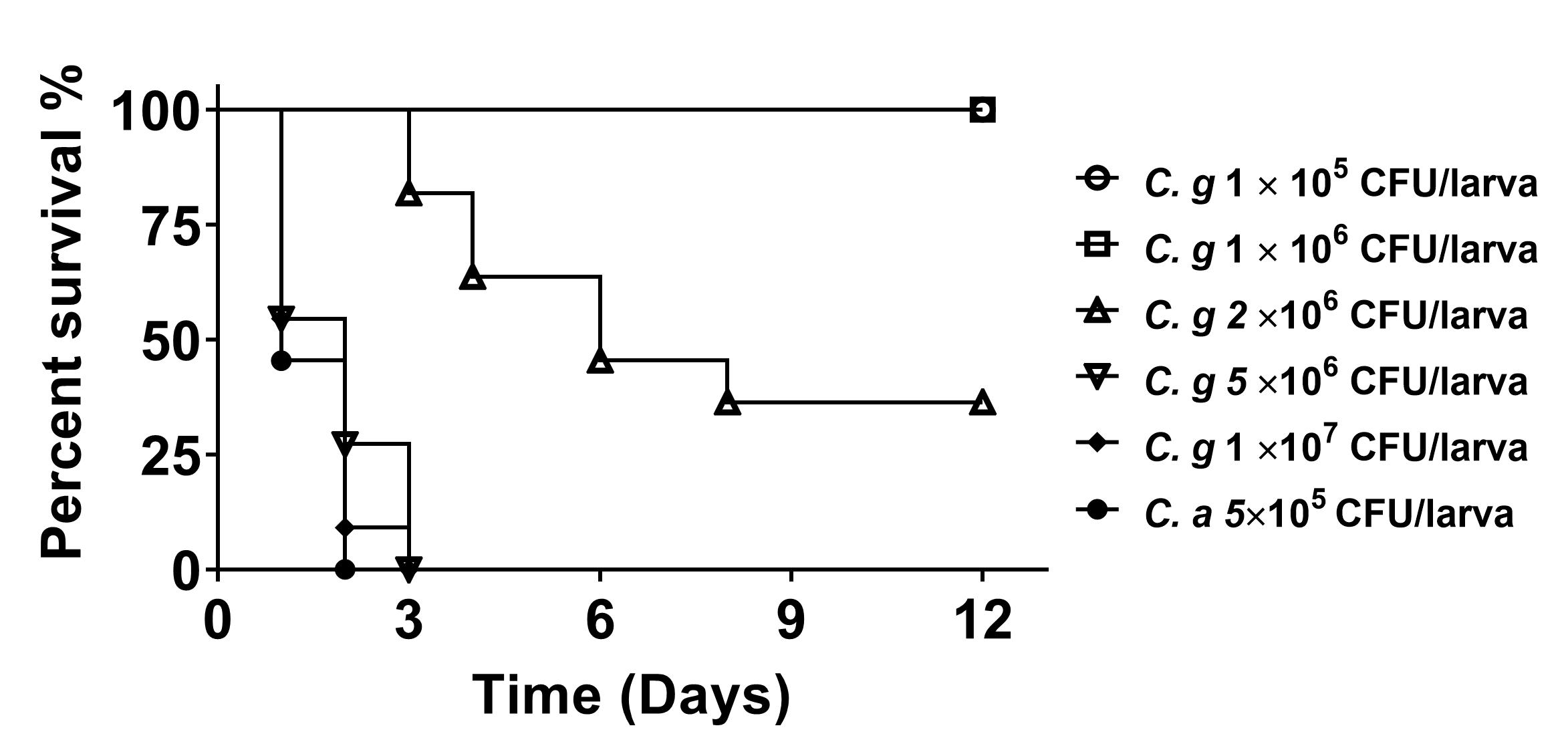

Supplement: Supplemental Material [file KVIR_A_1848107_SM7405.zip › S1.jpg]
